# Cost-effectiveness of diagnostic tools and strategies for the screening and diagnosis of tuberculosis disease and infection: a scoping review

**DOI:** 10.1136/bmjph-2023-000276

**Published:** 2024-04-19

**Authors:** Tom Ockhuisen, Alexandra de Nooy, Helen E Jenkins, Alvin Han, Colin A Russell, Shaukat Khan, Sarah Girdwood, Morten Ruhwald, Mikashmi Kohli, Brooke E Nichols

**Affiliations:** 1 Medical Microbiology, Amsterdam UMC Locatie Meibergdreef, Amsterdam, The Netherlands; 2 Department of Medical Microbiology, Amsterdam University Medical Center, Amsterdam, The Netherlands; 3 Department of Biostatistics, Boston University School of Public Health, Boston, Massachusetts, USA; 4 FIND, Geneva, Switzerland

**Keywords:** Public Health, Comorbidity, Communicable Disease Control, Mass Screening

## Abstract

The objective of this scoping review is to understand the cost-effectiveness of current and future tools/strategies for screening and diagnosis of tuberculosis (TB) infection and disease. To this end, PubMed, EMBASE and SCOPUS were used to identify any English language reports on the cost-effectiveness of TB infection/disease screening/diagnostic strategies published between 1 January 2017 and 7 October 2023. Studies included high-burden/risk TB populations, compared diagnostic/screening methods and conducted a cost-effectiveness/economic evaluation. We stratified the included articles in four groups (cost-effectiveness of diagnosing TB disease/infection and cost-effectiveness of screening for TB disease/infection). A full-text review was conducted, and relevant costing data extracted. Of the 2417 articles identified in the initial search, 112 duplicates were removed, and 2305 articles were screened for title and abstract. 23 full articles were reviewed, and 17 fulfilled all inclusion criteria. While sputum smear microscopy (SSM) has been the primary method of diagnosing TB disease in high-burden countries, the current body of literature suggests that SSM is likely to be the least cost-effective tool for the diagnosis of TB disease. Further scale-up with molecular diagnostics, such as GeneXpert and Truenat, was shown to be broadly cost-effective, with a multitest approach likely to be cost-effective for both screening and diagnosis. There is an urgent need to increase access and remove barriers to implementation of diagnostics that have been repeatedly shown to be cost-effective, as well as to develop new diagnostic and screening technologies/strategies to address current barriers to scale-up.

## Introduction

 Tuberculosis (TB) is a leading cause of ill health worldwide with a disproportionate impact on low-income and middle-income countries (LMICs) and marginalised communities in high-income settings.^1^ The 30 high TB-burden countries, all of which LMICs, represent 86% of all incident cases globally.^2^ Missed cases and untreated TB have consequences for both clinical care at the individual level, and for onward transmission of infection. Annually, around 30% of people with TB are not detected at all, hampering efforts for global TB control. Missed diagnosis is often a result of economic, geographical and health system barriers to accessing effective TB care.^3 4^

To reduce the global TB burden, modelling studies suggest improving uptake of TB treatment.^5 6^ Delay in uptake of treatment constitutes a major barrier to effective TB management.^7^ To effectively initiate people with TB onto treatment, accurate and cost-effective screening and diagnostic strategies are crucial.

Sputum smear microscopy (SSM) and culture as standalone techniques have been treated as the reference standard for decades when diagnosing TB. Both of these diagnostics have their limitations—sputum culture takes a relatively long time to provide results (up to 8 weeks),^8^ is costly, requires trained personnel and strong infrastructure and is therefore inaccessible to most primary clinics.^9^ SSM has low sensitivity compared with culture and also requires trained laboratory technicians to perform the test.^9 10^ In 2010, the WHO recommended the use of a new diagnostic, GeneXpert MTB/RIF, which detects TB as well as rifampicin resistance within 2 hours as the primary diagnostic in high-burden countries for people with presumed TB disease.^11^ Although more sensitive than SSM, it is significantly more expensive, which has been one of several implementation and scale-up challenges in LMICs.^12 13^

Classically, TB was thought to be dichotomous (infection vs disease), but currently, we know that progression through infection to symptomatic disease is better described as a continuum.^14^ However, management of TB remains tailored to only two states: TB infection and TB disease.^15^ Pulmonary TB disease is characterised by at least one of four symptoms: cough, fever, night sweats and weight loss.^16^ People with TB infection do not present with any disease symptoms, but are at risk of eventual progression to subclinical (asymptomatic, but bacteriologically confirmed)^17^ or symptomatic TB disease.^18^ Due to the different nature of TB infection and disease, their diagnosis, screening methods and subsequent cost-effectiveness differ as well. TB infection can be diagnosed with a tuberculin skin test (TST) (although there is cross-reactivity with prior BCG vaccination) and whole-blood interferon-gamma assay (IGRA).^19^ TB disease is primarily diagnosed by SSM, GeneXpert/other nucleic acid amplification tests and chest X-ray (CXR). Moreover, testing for the detection of the lipoarabinomannan (LAM) antigen in urine has emerged as a diagnostic. Its sensitivity is suboptimal compared with traditional diagnostics; however, it has demonstrated improved sensitivity for TB diagnosis in people coinfected with HIV.^20^

The WHO recommended screening for TB disease through four-symptom screening, CXR or molecular rapid diagnostics as standalone techniques or in combination.^21^ Rapid diagnostics consist of GeneXpert, Truenat and LAMP, and their usage is dependent on country-specific resources.^22 23^ TB infection is screened in the same way as it is diagnosed: through TST and IGRA. Although screening and diagnosis have their similarities in terms of tests used, screening tests are not to be regarded as diagnostics, and people with positive screening results should be further evaluated depending on the screening algorithm used.^21^

Here, we aim to carry out a scoping review to understand the cost-effectiveness of current and future tools/strategies for screening and diagnosis of TB disease and infection. The results of this review will inform future research on cost-effectiveness of potential novel diagnostics and optimised screening strategies in high-burden settings.

## Methods

The objective of this scoping review is to understand the cost-effectiveness of TB diagnosis and screening strategies in high-burden settings (online supplemental table 1). High-burden settings are characterised by settings (countries, regions, communities) with a significantly high number of TB incident cases relative to its population size. Given the differences in cost-effectiveness by TB disease/infection and diagnosis in opposition to screening, we stratified screening and diagnosis by four subsections, depicted in table 1 below.

**Table 1:** Four subsections stratified by identification type (four subsections covered in the study stratified by the tuberculosis nature and identification manner)

Diagnosis	Screening
The cost-effectiveness of diagnosing tuberculosis infection	The cost-effectiveness of screening for tuberculosis infection
The cost-effectiveness of diagnosing tuberculosis disease	The cost-effectiveness of screening for tuberculosis disease

### Search strategy and study selection

Studies on the cost-effectiveness of different TB diagnostics and screening strategies in high-burden settings from health databases, including PubMed, EMBASE and SCOPUS, were identified. The Medical Subject Headings (MeSH) database on PubMed was used to structure search terms, all in the English language (online supplemental table 2). Databases that did not support MeSH-controlled search algorithms were searched by slight alterations of the MeSH term. Search terms are provided in the online supplemental data. Articles published in the last 5 years—January 2017 and October 2023—were considered for inclusion, because studies within this period provide the most recent information on the cost-effectiveness of TB diagnostics and screening strategies. The final search was conducted on 7 October 2023.

Citations were imported into EndNote citation manager and de-duplicated. Review of all titles and abstracts of the resulting de-duplicated articles was conducted by two study team members (TO and AdN) and assigned a reason for inclusion or exclusion using Rayyan QCRI software.^24^ Any initial title/abstract where there was uncertainty of inclusion was jointly reviewed by an additional reviewer (BEN). A full-text review was then conducted of included articles (TO and AdN), and any articles where inclusion/exclusion was further uncertain were again jointly reviewed with an additional reviewer (BEN).

### Data extraction and analysis

The data extraction tool included information on country/target population, sample size, outcome time frame, outcome type, payer perspective and information required to identify which subsection (from table 1) the article should be assigned to.

To improve result synthesis, full outcomes were stratified by subsection. Data extracted from these comparisons include outcome measure (eg, cost per disability-adjusted life year averted and cost per life year saved), outcome values by strategy (as reported in the respective manuscripts) and willingness-to-pay threshold (if indicated). Where possible, outcomes were compared within each subsection and with respective willingness-to-pay thresholds (where available).

### Patient and public involvement

Patients and the public were not involved in this manuscript. This scoping review predominantly uses simulated cohorts and empirical outcome data from the literature.

## Results

### Sources identified

**Figure 1:**
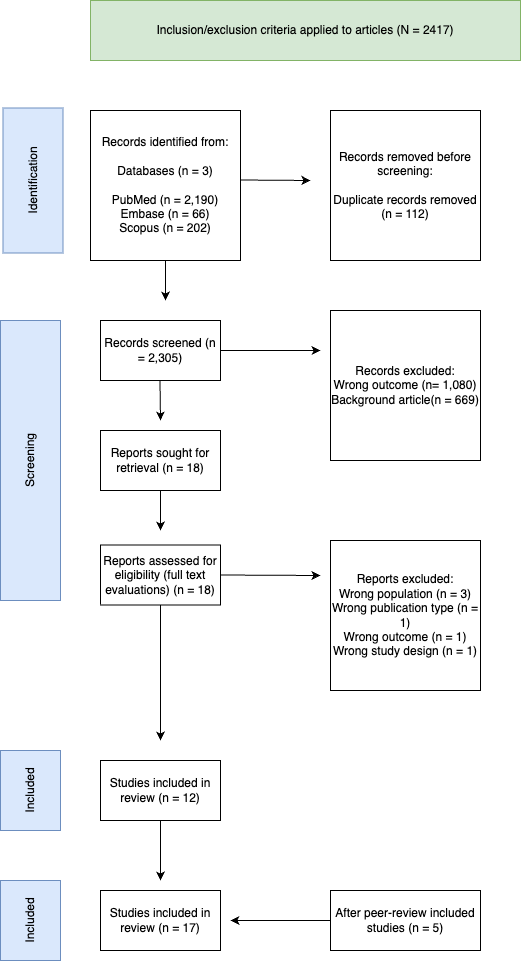
The Preferred Reporting Items for Systematic Reviews and Meta-Analyses diagram for the scoping review,^40^ including reasons for exclusion. A total of 2417 articles were found using the above-mentioned search terms, and 112 duplicates were removed, resulting in 2305 articles screened for title and abstract. Studies were included if: (1) the study population concerned a high-burden/risk tuberculosis group; (2) diagnostics or screening methods were compared; and (3) a cost-effectiveness/economic evaluation was conducted. Examples of studies excluded at this stage consisted of reviews (wrong publication type), studies with low-burden settings (wrong population) and other reasons depicted in figure 1. We did not exclude studies based on cofactors (HIV status) or other patient-specific characteristics.

23 studies were retrieved for full-text evaluations. After critical evaluations, 6 of these 23 articles were excluded on the basis of the criteria described above and in figure 1. Articles were excluded because of low-burden settings (n=3), wrong publication type (n=1), wrong outcome (n=1) or wrong study design (n=1). We included studies that made use of primary data/costs from other studies. A total of 17 studies were included in this review.

### Included studies

The 17 studies included in this review were conducted in 14 countries (Brazil, Mozambique, Kenya, Malawi, Thailand, India, South Africa, Tanzania, Zimbabwe, Zambia, Uganda, the Philippines, Vietnam and a native community in Canada) (tables2  3). This included the cost-effectiveness of screening tests/strategies (7 studies) and diagnosis (10 studies). Of these 17 studies, 15 evaluated the cost-effectiveness of TB disease diagnosis/screening strategies while only 2 evaluated TB infection. All included studies used a healthcare provider costing perspective, and roughly half were among people living with HIV (PLHIV) or severely immunocompromised individuals.

**Table 2:** Summary of the main characteristics of reviewed studies on the cost-effectiveness of diagnosing TB disease and infection

Source	Country	Study aim	Study design	Study population
**Diagnosing TB disease**				
Yakhelef *et al*^41^	Kenya	Evaluate the cost-effectiveness of introducing urine LF-LAM in different TB diagnostic algorithms	Decision analysis tree	Adults (>15 years) with TB symptoms hospitalised in the inpatient department (severely ill or immunosuppressed PLHIV)
Pooran *et al*^42^	South Africa	Determine the cost-effectiveness of five testing strategies for pleural TB diagnosis in South Africa	Decision tree model	A cohort of 1000 suspected TB cases presenting at primary clinics—suspected of pleural TB
Chitpim *et al*^26^	Thailand	Evaluate the cost-utility of molecular TB diagnostics in the general population suspected of pulmonary TB	Hybrid decision tree Markov model	The general population (>15 years of age) who presented with symptoms related to pulmonary TB or had an abnormal chest X-ray
Reddy *et al*^28^	South Africa and Malawi	Evaluate the cost-effectiveness of FujiLAM for TB testing	Microsimulation model	Hospitalised PLHIV
Lee *et al*^43^	India	Project the costs, cost-effectiveness and clinical impact of Truenat as replacement for smear microscopy or GeneXpert	Monte Carlo state-transition model	A cohort (>15 years of age) of adult, HIV-negative patients with presumptive pulmonary TB
Pooran *et al*^44^	South Africa, Zambia, Zimbabwe and Tanzania	Determine the cost-effectiveness of deploying point-of-care GeneXpert relative to smear microscopy	Clinical trial	A cohort of 1502 participants (>18 years of age) with presumptive TB recruited from four southern African countries
Vassall *et al*^27^	South Africa	Evaluate the cost-effectiveness of GeneXpert compared with SSM in the real world during national roll-out	Pragmatic trial	4656 individuals (>18 years of age) and able to provide a sputum sample were enrolled and followed up for 6 months
Thompson *et al*^45^	Uganda	Evaluate the cost-effectiveness of the Xpert Performance Evaluation for Linkage to Tuberculosis Care Study strategy	Pragmatic trial	4867 women and 3139 men all >18 years of age presenting for TB diagnostic care
Fekadu *et al*^46^	South Africa	Evaluate the cost-effectiveness of sputum-based Xpert tests with and without urine-based LAM assays	Decision analytical tree model	A hypothetical cohort of adult HIV-infected individuals with signs and symptoms of TB
**Diagnosing TB infection**				
Steffen *et al*^31^	Brazil	Evaluate the cost-effectiveness of newer TB infection diagnostic tests relative to the standard of care in PLHIV	State-transition Markov model	A cohort of PLHIV with a CD4^+^ cell count of 350 cells/µL or greater

LAMlipoarabinomannanLF-LAMlateral flow urine lipoarabinomannanPLHIVpeople living with HIVSSMsputum smear microscopyTBtuberculosis

**Table 3:** Summary of the main characteristics of reviewed studies on the cost-effectiveness of screening strategies for TB disease and infection

Source	Country	Study aim	Study design	Study population
**Screening for TB disease**				
Orlando *et al*^38^	Mozambique	Evaluate the cost-effectiveness of screening protocols in PLHIV to improve pulmonary case detection	Decision tree model	A cohort of 1000 HIV-positive antiretroviral treatment-naïve patients
Reddy *et al*^47^	Malawi and South Africa	Project the clinical and economic outcomes and cost-effectiveness of urine-based TB screening	Microsimulation model	A cohort of 1 000 000 hospitalised PLHIV
MacPherson *et al*^9^	Malawi	Evaluate the costs and yield from HIV-TB screening, including AI-aided chest X-ray	Pragmatic randomised trial	Adults (>18 years old) who reported cough of any duration (excluding individuals who had taken TB treatment in the preceding 6 months)
Kim *et al*^32^	South Africa	Assess yield of TB cases using GeneXpert and the cost-effectiveness of adding digital CXR to symptom screening	Retrospective within trial	A total of 61 580 inmates were screened for symptoms
Datta *et al*^33^	India	Assess the yield and cost of two mobile digital CXR strategies to detect pulmonary TB	Observational	In strategy one: adults (>18 years old), who were eligible, but did not receive CXR (n=13)In strategy two: all adults with chest symptoms (n=50)
Brümmer *et al*^48^	India, South Africa, the Philippines, Uganda and Vietnam	Evaluate the ability of screening tests to make community-based case-finding cost-effective	Microsimulation model	Each screening strategy was simulated in a hypothetical cohort of 100 000 individuals (>15 years of age) in a community setting
**Screening for TB infection**				
Uppal *et al*^35^	Canada (indigenous communities)	Evaluate the cost-effectiveness of community-wide active TB infection screening	Decision analysis model	Two Inuit villages with a collective population of 2500 people

AIartificial intelligenceCXRchest X-rayPLHIVpeople living with HIVTBtuberculosis

### Cost-effectiveness of diagnosing TB disease

From the 17 papers that met our selection criteria, 9 covered diagnosis of TB disease and were performed in Kenya, South Africa, Thailand, India, Malawi, Uganda, Zambia, Zimbabwe and Tanzania. Among these studies, similar diagnostic tools were compared, such as GeneXpert+LAM versus GeneXpert alone and GeneXpert versus SSM, and could therefore be compared (table 4). Willingness-to-pay thresholds were not reported by three out of nine studies.

**Table 4:** Comparison between results from studies reporting on the cost-effectiveness of diagnosing for TB disease and infection

Study	Country	**Diagnostic strategies evaluated**	Outcome measure	**Outcome**	WTP	Conclusions
**Diagnosing TB disease**						
Yakhelef *et al*^41^	Kenya	GeneXpert+LAM vs GeneXpert LAM vs SSM SSM+LAM vs SSM SSM+LAM vs SSM+CXR LAM+SSM+CXR vs SSM+CXR SSM+LAM vs GeneXpert GeneXpert+GeneXpert urine vs GeneXpert LAM+GeneXpert vs GeneXpert+GeneXpert urine LAM+GeneXpert vs GeneXpert+CXR LAM+GeneXpert+CXR vs GeneXpert+CXR	Cost perDALY averted	$24 per DALY averted for LAM+GeneXpert compared with GeneXpert alone LAM cost-saving compared with SSM $16.42 per DALY averted for SSM+LAM compared with SSM $93.2 per DALY averted for SSM+LAM vs SSM+CXR $7.9 per DALY averted for LAM+SSM+CXR vs SSM+CXR $39.05 per DALY averted for SSM+LAM vs GeneXpert $102.5 per DALY averted for GeneXpert+GeneXpert_urine vs GeneXpert LAM+GeneXpert cost-saving compared with GeneXpert+GeneXpert_urine LaAM+GeneXpert dominated by GeneXpert+CXR $12.3 per DALY averted for LAM+GeneXpert+CXR compared with GeneXpert+CXR	$3106	First, replacing smear microscopy with LF-LAM test is highly cost-effective.Second, it is also cost-effective to add LF-LAM testing in addition to smear.Third, it is more cost-effective to perform smear and LF-LAM tests than GeneXpert test alone and when GeneXpert is available, it is more cost-effective to perform GeneXpert in sputum and LF-LAM test rather than GeneXpert in sputum and GeneXpert in urine.
Reddy *et al*^28^	SouthAfrica andMalawi	GeneXpert+LAM vs GeneXpert GeneXpert+AlereLAM vs GeneXpert+FujiLAM	Cost per life years saved (LYS)	$830 per LYS in South Africa (GeneXpert+LAM compared with GeneXpert alone)$440 per LYS in Malawi (GeneXpert+LAM compared with GeneXpert alone)**South Africa:** GeneXpert+AlereLAM: dominatedGeneXpert+FujiLAM: $830 per LYS **Malawi:** GeneXpert+AlereLAM: dominated GeneXpert+FujiLAM: $440 per LYS	NA	GeneXpert+LAM is more cost-effective in Malawi than in South Africa, but is cost-effective compared with GeneXpert alone.GeneXpert+FujiLAM is cost-saving compared with GeneXpert+AlereLAM.
Chitpim *et al*^26^	Thailand	SSM vs GeneXpert TB-LAMP vs SSM TB-LAMP in addition to SSM	Cost perQALY gained	$6.16 per QALY gained for GeneXpert compared with SSM TB-LAMP compared with SSM: dominated TB-LAMP as an add-on to SSM: $28.40 per QALY gained	$5008	GeneXpert is more cost-effective as initial or add-on tests than SSM with culture.TB-LAMP as a second test alongside GeneXpert is cost-effective.
Pooran *et al*^42^	South Africa	A comparison of SSM, Xpert Ultra, MGIT (Mycobacterial-Growth-In-Tube liquid culture), ADA (adenosine deaminase) and IRISA-TB (unstimulated interferon-gamma) for diagnosis of pleural TB.	Cost per pleural TB case diagnosed and initiated on treatment	SSM: $1 262 935 per case diagnosed and initiated on treatmentXpert Ultra: $94 633MGIT: $85 914ADA: $49 065IRISA-TB: $44 084	NA	IRISA-TB is the most cost-effective strategy for diagnosing pleural TB.Biomarker-based diagnostic strategies (ADA, IRISA-TB) were more cost-effective than the microbiological tests.
Lee *et al*^43^	India	SSM vs Truenat (in designated microscopy centres (DMC) or point of care (POC))GeneXpert vs Truenat (DMC and POC)	YLS	Truenat DMC vs SSM: $240 per YLS Truenat DMC vs GeneXpert: weakly dominated Truenat POC vs SSM: $210 per YLS Truenat POC vs GeneXpert: $120 per YLS	$990	Truenat DMC is cost-effective compared with SSM, but was slightly outperformed by GeneXpert.Truenat POC outperformed both SSM and GeneXpert.
Pooran *et al*^44^	South Africa, Zambia, Zimbabwe and Tanzania	SSM vs POC GeneXpert	ICER per case diagnosed by index test	Tanzania: $4254 per case diagnosed with GeneXpertZambia: GeneXpert was dominated by SSMZimbabwe: $1675 per case diagnosed with GeneXpertSouth Africa: $1373 per case diagnosed with GeneXpert	$9450	POC GeneXpert implementation is cost-effective compared with SSM. Transmission reduction and drug resistance detection were not factored into the analysis, meaning that the strategy is likely to be even more cost-effective.
Vassall *et al*^27^	South Africa	SSM vs GeneXpert	DALYs averted	3% discount rate: −0.09 DALYs averted for GeneXpert vs SSM	Wide range of WTP thresholds used	No evidence was found that GeneXpert improved the cost-effectiveness of TB diagnosis compared with SSM.
Thompson *et al*^45^	Uganda	Decentralised Xpert vs on-site SSM with specimen transport for centralised Xpert	Cost per additional TB case diagnosed and cost per additional treatment initiation in 14 days	$1332 per additional TB case diagnosed for decentralised testing compared with centralised$687 per additional treatment initiation in 14 days	NA	Costs were similar between decentralised and centralised testing, but decentralised testing resulted in more patients receiving appropriate Xpert testing, resulting in a higher per-patient cost.
Fekadu *et al*^46^	South Africa	SSM vs Xpert Ultra vs Xpert Ultra+LAM	Cost per DALY averted	SSM vs Xpert Ultra: $71.4 per DALY avertedXpert Ultra vs Xpert Ultra+LAM: $676.9 per DALY averted	$202 per DALY averted	Xpert Ultra+FujiLAM is cost-effective in HIV-infected individuals (compared with SSM).
**Diagnosing TB infection**						
Steffen *et al*^31^	Brazil	New skin tests compared with TST	Cost per QALY gained	EC skin test: dominatedDiaskintest: cost-savingQFT-plus: dominated	$7544	The Diaskintest was cost-saving across all tests, but all newer tests are cost-saving compared with TST.

CXRchest X-rayDALYdisability-adjusted life yearICERincremental cost-effectiveness ratioLAMlipoarabinomannanLF-LAMlateral flow urine lipoarabinomannanNAnot availableQALYquality-adjusted life yearSSMsputum smear microscopyTBtuberculosisTSTtuberculin skin testWTPwillingness to pay

In general, GeneXpert in combination with LAM was found to be more cost-effective than GeneXpert alone (table 4). The power of a LAM test is enhanced when used in combination with other diagnostics such as GeneXpert or SSM.^25^ However, the use of LAM as a primary tool for diagnosis will be limited by the target group, given that LAM is most effective and recommended to diagnose TB disease in PLHIV.

Moreover, Truenat outperformed both GeneXpert and SSM as standalone techniques (table 4). Truenat point of care (POC) became more cost-effective than GeneXpert and SSM after 4 and 6 years, respectively. POC diagnostics greatly increase accessibility to testing, as decentralisation of diagnostics is playing a more prominent role in high-burden countries to reach more people; the comparison between Truenat POC and GeneXpert can provide important insights into how to frame new policies regarding diagnostic scale-up in the peripheral healthcare setting in high-burden countries.

SSM as a standalone diagnostic is rarely considered cost-effective in comparison with other available diagnostics. A two-test algorithm that includes SSM as well as either LAM or GeneXpert can, however, be cost-effective^26^ (table 4). GeneXpert is found to be generally more cost-effective than SSM. Even though the cost of GeneXpert is higher than SSM, costs can be saved through fewer false positives incorrectly initiating treatment, and fewer false negatives that continue to transmit TB to their contacts. However, an empirical study assessing the national roll-out of GeneXpert in South Africa concluded that there was no evidence GeneXpert improved the cost-effectiveness of diagnosing TB disease compared with SSM.^27^ This is partly due to the high rates of empirical treatment in spite of a negative smear result. This difference in findings highlights the importance of empirical cost-effectiveness analysis in addition to modelled cost-effectiveness estimates, and in the use of this type of empirical work to understand the potential barriers to the roll-out of new technology that may impede real-world cost-effectiveness. These findings also highlight the importance of understanding the true status quo, rather than the per-guideline status quo when conducting modelling analyses.

While molecular diagnosis of TB is broadly considered to be cost-effective, a notable exception to this is in the case of diagnosing non-pulmonary TB. In the case of pleural TB, biomarker-based tests were more cost-effective than microbiological tests.^28^

### Cost-effectiveness of diagnosing TB infection

From the 17 studies that met our selection criteria, 1 covered the cost-effectiveness of diagnosing TB infection. Steffen *et al* assessed the cost-effectiveness of implementing newer TB infection diagnostic skin tests in the standard of care for PLHIV in Brazil.

IGRAs such as the Diaskintest contain antigens absent in the BCG vaccine; therefore, performing IGRAs in BCG-vaccinated population has superior specificity than TSTs.^29 30^ IGRAs, compared with TSTs, were found to be cost-effective^31^ (table 4).

### Cost-effectiveness of screening for TB disease

From the 17 papers that met our selection criteria, 6 assessed the cost-effectiveness of screening for TB disease and were performed in Malawi, South Africa, India, the Philippines, Vietnam, Uganda and Mozambique. Among these studies, three compared a similar screening strategy: symptom screening (2) or SSM (1), followed by CXR. Four studies stated a willingness-to-pay threshold and only two studies used the same outcome measure (cost per TB case detected).

In general, the four-symptom screening followed by CXR was not found to be cost-effective (table 5). This differs slightly, however, depending on the underlying disease prevalence. The number of people who must screen positive to identify one correct TB diagnosis is likely too high for these low-sensitivity screening strategies. Additionally, one study first screened prisoners with four-symptom screening, but rather than only using CXR on symptom-positive individuals, CXR was performed on every individual being screened, significantly driving up costs.^32^ Importantly, the combination in which tests are used matters, for example, CXR as a screening tool followed by SSM as diagnostic was found to be more cost-effective than SSM (negative) followed by CXR.^33^ Furthermore, screening techniques using TB-LAM are more cost-effective than GeneXpert for TB screening.

**Table 5:** Comparison between results from studies reporting on the cost-effectiveness of screening for TB disease and infection

Study	Country	Diagnostic strategies evaluated	Outcome measure	Outcome	WTP	Conclusions
**Screening for TB disease**						
MacPherson *et al*^9^	Malawi	HIV-TB screening—CXR (and GeneXpert for abnormal CXR results) vs the standard of care(SOC)—SSM+Xpert	Cost perQALY gained	HIV-TB screening vs SOC for people with a cough:$4620.47 per QALY gained	$400 perQALY, $800 perQALY and $1200per QAL Y	Screening for TB among people with a cough is not cost-effective.
Kim *et al*^32^	South Africa (prison)	CXR as follow-up to symptom screening	Cost per TB case detected	The cost of following up symptom screening with CXR: $22 278 per additional TB case detected	NA	Additional TB cases were detected, but costs were high. This does vary with underlying prevalence.
Datta *et al*^33^	India	SSM+mobile CXR Mobile CXR+SSM	Cost per TB case detected	$32 per TB case detected $8 per TB case detected	NA	CXR followed by SSM is more cost-effective than vice-versa.
Orlando *et al*^38^	Mozambique	Four-symptom screening and SSM (standard) vs MTB/RIF Four-symptom screening and SSM (standard) vs LF-LAM/MTB/RIF	Cost per DALY saved	Standard vs MTB/RIF: $56.54 per DALY saved Standard vs LF-LAM/MTB/RIF: $72.31 per DALY saved	Very cost-effective: <$382Cost-effective: <$1146	MTB/RIF could lead to increased TB case detection and a reduction of costs compared with the standard and LF-LAM/MTB/RIF.
Reddy *et al*^47^	South Africa and Malawi	GeneXpert vs GeneXpert+TB- LAM	Cost per years of life saved (YLS)	**South Africa:** strategy (1) vs (2): $840 per YLS**Malawi:** strategy (1) vs (2): $450 per YLS	Cost-effective: <$750 per YLS in Malawi and <$940 per YLS in South Africa	Urine-based screening of patients living with HIV can be cost-effective in resource-limited settings.
Brümmer *et al*^48^	India, South Africa, the Philippines, Uganda and Vietnam	POC CRP screening (A) Hypothetical screen (95% sensitivity and 70% specificity) with cost equivalence to Xpert (B) Hypothetical screen (95% sensitivity and 70% specificity) with costs similar to CRP (C) Hypothetical screen, specificity increased to 95% with costs similar to CRP Xpert Ultra	Cost per DALY averted	**POC CRP screening:**India: $1300 per DALY avertedSouth Africa: $1300 per DALY avertedThe Philippines: $550 per DALY avertedUganda: $1200 per DALY avertedVietnam: $1500 per DALY averted**Hypothetical screen A:**India: $1500 per DALY avertedSouth Africa: $1700 per DALY avertedThe Philippines: 740 per DALY avertedUganda: $1700 per DALY avertedVietnam: $2200 per DALY averted**Hypothetical screen B:**India: $1100 per DALY avertedSouth Africa: $1100 per DALY avertedThe Philippines: $490 per DALY avertedUganda: $1100 per DALY avertedVietnam: $1300 per DALY averted**Hypothetical screen C:**India: $780 per DALY avertedSouth Africa: $920 per DALY avertedThe Philippines: $390 per DALY avertedUganda: $770 per DALY avertedVietnam: $940 per DALY averted**Xpert Ultra:**India: $1500 per DALY avertedSouth Africa: $1500 per DALY avertedThe Philippines: $670 per DALY avertedUganda: $1600 per DALY avertedVietnam: $2000 per DALY averted	India: $560 per DALY avertedSouth Africa: $3700 per DALY avertedThe Philippines: $1100 per DALY avertedUganda: $190 per DALY avertedVietnam: $710 per DALY averted	Low-complexity screening tools can achieve meaningful improvements in TB diagnosis if they are highly sensitive, highly specific and available at low cost.
**Screening for TB infection**						
Uppal *et al*^35^	Canada (indigenous communities)	No active screening Community-wide screening once Community-wide screening every 2 years for 20 years	Cost per latent TB case averted	Dominated Comparator $4795 per active TB case averted	NA	Active community screening has the potential to be cost-saving in high TB-burden populations.

CRPC reactive proteinCXRchest X-rayDALYdisability-adjusted life yearLF-LAMlateral flow urine lipoarabinomannanNAnot availablePOCpoint of careQALYquality-adjusted life yearSSMsputum smear microscopyTBtuberculosisWTPwillingness to pay

### Cost-effectiveness of screening for TB infection

From the 17 studies that met our selection criteria, 1 covered screening for TB infection (table 5). Uppal *et al* assessed the cost-effectiveness of active screening for people with TB infection and TB disease in indigenous communities in Canada.

Diagnosis and treatment of TB infection cases are essential in reducing the future pool of active TB cases by early identification, especially among high-risk groups.^34^ In a modelling study of community-wide screening in native communities in Canada^35^ (high-risk group), biennial screening with TST and CXR (for active cases) over a 20-year period was cost-effective and helped reduce the TB burden compared with no active screening.^35^ This result adds to the already increasing awareness that screening for and treatment of TB infection in high-risk groups should be part of primary care cascades, especially when aiming for global control and the elimination of TB.^36^

## Discussion

Our review aimed to assess the cost-effectiveness of current tools for the screening and diagnosis of TB disease and infection. While SSM has been the primary method of diagnosing TB disease in high-burden countries, the method has been continually demonstrated to be the least cost-effective tool in multiple studies, outperformed by GeneXpert and urine-based tests (LAM). While there is a growing body of literature on which diagnostics and screening strategies may be cost-effective and impactful, they are not implemented across all countries and subpopulations within countries. These results support a call to action to either determine pathways to access the most cost-effective diagnosis and screening strategies, or to develop novel diagnostic/screening strategies that overcome the current limitations to scale-up of these existing diagnostics. Furthermore, the relative contribution of subclinical TB on disease transmission is increasingly studied; however, there are no cost-effectiveness studies to date quantifying the potential cost-effectiveness of existing or novel diagnostics for detecting subclinical TB.

In general, a multitest approach is cost-effective for screening and diagnosis. Using multiple diagnostics aids in improving pretest probabilities and ultimately leads to fewer people incorrectly initiated on treatment. Moreover, the order in which diagnostic/screening tests are used matters for impact and cost-effectiveness. For example, CXR followed by SSM had a larger impact and was more cost-effective than SSM followed by CXR.^33^

Screening for TB disease is usually performed through four-symptom screening followed by CXR; this follow-up ensures improved sensitivity of four-symptom screening.^37^ CXR as a standalone screening method is more effective than four-symptom screening, but also more expensive. Multiple studies assessed the cost-effectiveness of different follow-up tests for four-symptom screening.^9 32 33 38^ Screening was generally not considered very cost-effective unless pretest probability was high. Better screening strategies are needed to fix this, and assessment of other screening strategies recommended by the WHO, such as computer-aided detection,^39^ can be valuable.

Screening and diagnosis for TB infection are likely to be most cost-effective in high-burden countries. Screening and diagnosis can also be even more cost-effective when targeted to subpopulations which are known to have high TB prevalence within these countries—or even high-risk groups within low-burden countries (such as Canada with a low overall burden at a national level, but high TB prevalence among indigenous communities).

This scoping review has several limitations. First, results on cost-effectiveness of diagnostics and screening strategies for TB infection were not easily generalisable due to the small number of studies reporting on this. While the breadth of literature on the topic is lacking, some important results of these studies remain clear, such as the importance of underlying disease prevalence in the cost-effectiveness of diagnostic/screening strategies. Second, the majority of the included studies are results from mathematical models. While these questions of cost-effectiveness can be effectively analysed using mathematical modelling, empirical confirmation would be useful, particularly to understand the cost-effectiveness of different diagnostic/screening strategies as implemented on the ground in different contexts. Third, the baseline diagnostics or screening strategies are heterogeneous between the studies included in this review, which makes comparison between studies more difficult. Despite this, broad conclusions could be drawn in the context of respective baseline diagnostic/screening strategies. Fourth, since we have opted for a scoping review, we have not provided critical appraisal of the economic analyses that were undertaken. We have instead aimed to provide a broad current state of the cost-effectiveness literature for TB screening and diagnosis.

Through this scoping review, we have identified a number of gaps in the literature. While multiple diagnostic and screening strategies have been found to be cost-effective in their respective studies, literature on implementing these strategies in real-world settings, or studies looking across multiple settings, is limited. Moreover, the potential cost-effectiveness of new or future diagnostic tools is under-represented in the current body of literature, even though these can be of great value in guiding policy and implementation once these new diagnostics are available. Furthermore, only a small number of studies report on the combination of multiple already existing diagnostics for screening and diagnosing TB disease and infection; additional literature and understanding in this area can be of great value for reducing national and global TB burden in the short term.

## Conclusion

To conclude, given the currently available tools, further scale-up of molecular diagnostics will continue to be considered cost-effective, with a multidiagnostic approach likely to be cost-effective for both screening and diagnosis in many settings. All published studies have been based on existing technologies, and the cost-effectiveness of potential novel diagnostic and screening strategies is unknown. Novel diagnostics that either improve access or accuracy, or are less expensive than the current molecular diagnostics, are likely to be considered cost-effective. There is an urgent need to increase access to and remove barriers to implementation of diagnostics we know to be cost-effective, as well as develop new diagnostic and screening technologies or tools to address current barriers to scale-up.

## supplementary material

10.1136/bmjph-2023-000276 online supplemental file 1
