# Supplementary material for: Cost-effectiveness of diagnostic tools and strategies for the screening and diagnosis of tuberculosis disease and infection: a scoping review
Source: BMJ Public Health. 2024 Apr 19;2(1):e000276. doi: 10.1136/bmjph-2023-000276 (PMC11816851; doi:10.1136/bmjph-2023-000276)
Supplement: online supplemental file 1 [file bmjph-2-1-s001.pdf]

## Supplementary information

### *Diagnostics assessed*

**Supplementary table 1.** Table depicting different diagnostics, turn-around times, sensitivity and specificity.

| Diagnostic | Specimen type | Turn-around time | Sensitivity | Specificity | Source |
|---|---|---|---|---|---|
| GeneXpert | Sputum | <1 day | 88% | 99% | [44] |
| SSM | Sputum | <1day | 64% | 98% | [30,45] |
| Truenat | Sputum | <1 day | 86% | 99% | [30] |
| LAM | Urine | <4 hours | 50%* | 84.2%* | [46,47] |
| CXR (Digital) | - | 1 hour | 98% | 75% | [48,49] |
| LAMP | Sputum | 1 hour | 77.9% | 97.9% | [50,51] |
| TST | - | 2-3 days | 85% | 88% | [52,53] |
| IGRA | Blood | 1 day | 74% | 78% | [54,55] |

*Indicates that the value can differ respective of the target population

### *Search terms used in a variety of databases*

**Supplementary table 2.** Three databases with search terms used in this review

| **(1) PubMed** |
|---|
| ((((“Cost-Benefit Analysis/Economics” [MeSH] AND “diagnosis” [subheading]) OR “mass screening” [MeSH]) AND “Tuberculosis” [MeSH]) OR “latent tuberculosis” [MeSH]) |
| **(2) EMBASE** |
| ‘tuberculosis’/exp AND ‘cost-effectiveness analysis’/exp AND ‘screening’/exp OR ‘diagnosis’/exp |
| **(3) SCOPUS** |
| Cost-effectiveness analyses AND Tuberculosis AND diagnosis OR screening |
